# Temporal trends and spatial distribution of leishmaniasis based on biomes in Brazil, 2007 to 2020

**DOI:** 10.1590/S1678-9946202668044

**Published:** 2026-07-20

**Authors:** Geisa Bezerra Ferreira, Leonardo Augusto Kohara Melchior, Andreia Fernandes Brilhante

**Affiliations:** 1 Universidade Federal do Acre, Centro de Ciências da Saúde e do Desporto, Programa de Pós-Graduação em Ciências da Saúde na Amazonia Ocidental, Rio Branco, Acre, Brazil

**Keywords:** American cutaneous leishmaniasis, Visceral leishmaniasis, Amazon, Prais–Winsten method

## Abstract

Analyses based on biomes provide a less fragmented view than usual studies and help the understanding of epidemiological characteristics from different perspectives. We describe the temporal trends and spatial distribution of American cutaneous leishmaniasis (ACL) and visceral leishmaniasis (VL) in Brazilian biomes, from 2007 to 2020. This ecological survey used data from the Brazilian Notifiable Diseases Information System. The Prais–Winsten method was used for temporal analyses. ACL and VL incidences were calculated based on sex and age groups, relative risk considering these two variables, and mortality, lethality, cure, and treatment abandonment rates per biome. The Amazon, Atlantic Forest, Cerrado, and Caatinga biomes showed a decreasing temporal trend, varying from −1% to −3% for ACL and VL, respectively. Other biomes showed a stationary temporal trend for both diseases. VL had a similar transmission profile in all biomes, affecting children aged 0–4 years regardless of sex. In contrast, ACL mainly affected older adults, except in the Amazon and Pampa, where working-age males were most commonly affected. In all biomes, being a man constituted a risk factor for acquiring these diseases, abandoning treatment, and dying, whereas being a woman configured a factor that favored cure and protected against lethality. Our findings showed a decrease in ACL and VL incidence in most biomes and a stable incidence in other biomes. The results of our study showed that the distinct transmission profiles of ACL and VL have persisted across different biomes with minute differences.

## INTRODUCTION

Leishmaniases are a group of parasitic diseases caused by *Leishmania* species that are transmitted to humans and animals by the bite of infected female sandflies^1,2^. Brazil reported 91.1% of visceral leishmaniasis (VL) cases and 36.9% of American cutaneous leishmaniasis (ACL) in 2023, being one of the countries with the highest number of reported cases in Latin America^3^.

Cases have occurred in all Brazilian macroregions, requiring vector and reservoir control actions from epidemiological surveillance to contain their spread^3-5^. However, Brazil, a country of continental dimensions with a wide variety of vegetation, relief, and climate, has a rich biodiversity of fauna, with diverse hosts, vectors, and pathogenic agents^6-10^. The country has six biomes: Amazonia, Cerrado, Atlantic Forest, Caatinga, Pantanal, and Pampa, each with its own ecological and environmental characteristics. Leishmaniasis can present varying epidemiological transmission profiles, influenced by parasite, host, and vector distribution and the socioeconomic and cultural issues of each biome. Thus, understanding endemic diseases according to biomes can provide different perspectives, contributing to preventive and combative health policies compatible with the reality. Notably, the spread of ACL and VL goes beyond political–administrative boundaries, requiring a more panoramic view, such as by biomes^11,12^.

Spatial and temporal trend analyses have evaluated the spatiotemporal distribution of diseases^13^. Analyses based on biomes can offer a less fragmented view than that of usual studies; thus contributing to a better understanding of the epidemiological characteristics of a disease; providing insights to formulate disease prevention, control, and intervention strategies; improving public health decision-making^14,15^; and directing efforts to larger locations and group risks. Given the environmental and ecosystem differences between Brazilian biomes, this study aims to describe the temporal trends, spatial distribution, and epidemiological characteristics of ACL and VL based on Brazilian biomes from 2007 to 2020.

## MATERIALS AND METHODS

### Study area

Brazil, in South America, has the fifth-largest territorial area worldwide (8,515,759 km). It is home to approximately 213.3 million inhabitants across its 5,570 municipalities. Its topography, vegetation, biodiversity, and climate are distributed across the following six biomes: Amazon, Caatinga, Cerrado, Atlantic Forest, Pantanal, and Pampa^16^.

The Amazon, the largest biome in Brazil, encompasses its northern region and accounts for approximately 49% of its territory^10,17^. It is the world's largest tropical forest and sustains a great diversity of flora and fauna. Its area includes more than 1 million cataloged plant species, 3,000 fish species, 900 bird types, and various insects, reptiles, and mammals^10^. Furthermore, it has the largest hydrographic basin in the world, the river water of which is equivalent to 20% of the all freshwater reserves worldwide, which affects its humid equatorial climate^17,18^.

The Caatinga lies in northeastern Brazil, encompassing approximately 10% of the Brazilian territory^18^. During its dry period, the vegetation loses its leaves and the trunks become whitish. It has a rich biodiversity, with some species existing exclusively in this biome^10^. Caatinga has a semi-arid climate and two seasons (namely, dry and rainy)^19^ and experiences overall low rainfalls. Its short rainy season has torrential and irregular rain, whereas its dry or drought periods occur throughout the year^20^. This biome is most susceptible to desertification because of natural and anthropogenic factors, and the modification of its landscape puts the preservation of wild animals, water quality, and the balance of climate and soil at risk^10,20^.

Cerrado encompasses the entire Brazilian Central Plateau, occupying 24% of the Brazilian territory. It is recognized as the most abundant savannah in the world, with various plant types including herbaceous, shrubby, arboreal, and vine plants. Agricultural activities have used it since the mid-1960s due to its favorable vegetation cover. It has a rich fauna, and many vertebrate species only occur in this biome. However, its centralized geographic location and high biodiversity favor the exchange of species between biomes^21^. Its seasonal climate is characterized by dry winters and rainy summers, with well-defined annual temperature averages and rainfall^10^.

The Atlantic Forest has a humid tropical climate, with hygrophilous vegetation that forms several ecosystems. It extends along the Brazilian coast and occupies approximately 13% of the Brazilian territory. It accounts for approximately 27% of the native forest cover and is one of the richest biomes worldwide, with >1,300 species of Brazilian fauna, 567 of which are exclusive to this biome^10^. The wide distribution of its biota favors areas of endemism, with the presence of at least two endemic species with overlapping distributions that are preserved because of difficult access^22^.

The Pampa region is the smallest biome in Brazil, with an area equivalent to 2% of its territory. It has a characteristic rainy climate that has negative temperatures in winter, affecting fields and shrub vegetation^18^. Its rich flora and fauna had been reduced to effectively protected areas as conservation units^23^. Its typical vegetation includes fields with herbs and shrubs, and its plant uniformity facilitates natural pasture and agricultural activities^10^.

The Pantanal encompasses approximately 2% of Brazilian territory. A large proportion of Brazilian fauna occurs in this biome, with >1,100 species of flora and fauna. Compared with other biomes, it experiences a high degree of humidity owing to continuous floodplains and riparian forests, which are crucial for several phytophysiognomies^18^. The Pantanal vegetation resembles that of the Cerrado and Caatinga. During its rainy and flood periods, part of its fauna gathers in higher regions (named "cordilleras") — which normally experience no flooding, returning to their usual habitat under normalized water levels^24^.

### Design and data source

This ecological study was conducted using compulsory notification data from the Notifiable Diseases Information System, Ministry of Health, Brazil^25^. Confirmed and autochthonous cases were extracted from that system according to ACL and VL recurrence location, stratified by sex (male and female), age range (0–4, 5–19, 20–39, 40–59, and ≥60 years), case evolution (cure, abandonment, and mortality rates), and municipality of residence (located in the Amazon, Atlantic Forest, Cerrado, Caatinga, Pampa, or Pantanal).

To calculate incidence, demographic and socioeconomic information from TABNET and that regarding resident population from the study database of population estimates according to municipality, sex, and age, from 2007 to 2020, were obtained.

#### Data analyses

Disease incidence was calculated by biome, sex, age groups. The proportion between the number of cases and the population during the period was multiplied by 100,000 inhabitants.

For case evolution variables, cure $\left(\frac{\text{number of cured patients}}{\text{number of cases per year}}\right)$ , abandonment $\left(\frac{\text{number of abandonments}}{\text{number of cases per year}}\right)$ , and lethality rates $\left(\frac{\text{number of deaths}}{\text{number of cases per year}}\right)$ were calculated. All final values were multiplied by 100 to get the results in percentages (%). Additionally, the mortality coefficients for ACL and VL were calculated using the following formula: $\left(\frac{\text{total deaths from the disease per year}}{\text{the total population per year}}\times 100,000\;\text{inhabitants}\right)$ .

Some municipalities belong to a single biome. However, most (n=957) have political–administrative boundaries, making them belong to two or three biomes. Notably, the Brazilian Institute of Geography and Statistics finds that Brazil only has 5,570 municipalities.

Municipalities that belonged to more than one biome were excluded to analyze the incidences that were exclusive to each Brazilian biome (except for five municipalities in Pantanal that had smaller proportions of other biomes). Of the 5,570 municipalities, 4,611 were included for analysis. Of these, 435 municipalities were in the Amazon biome; 936, in the Caatinga; 760, in the Cerrado; 2,385, in the Atlantic Forest; and 90, in the Pampa. In the Pantanal, only Ladario shared no other biome. However, as one municipality is insufficient for analysis, those with at least 75% of their area in the Pantanal were included. For this, the vector function "intersection" from the Quantum Geographic Information System (version 3.22, QGIS Association, Laax, Swiss) and the Brazilian Institute of Geography and Statistics biomes and municipalities shapefiles were used. Finally, the municipalities of Ladario (100%), Barao de Melgaco (99.7%), Corumba (98.7%), Pocone (80.6%), and Aquidauana (79%) in the Pantanal were selected; the others were excluded from the analysis.

Temporal trends in Brazilian biomes were analyzed based on the number of cases to estimate disease trends, which included finding the occurrence of growth patterns, decline, and absence of trend (stationary) in the studied period. The annual percentage variation and percentage variation of cases per 95% confidence interval (CI) were also calculated.

Analyses were performed using the Prais–Winsten autoregressive analysis method on Stata 14, which accounts for first-order autocorrelation, providing the annual percentage increase rate and its respective 95%CI. The trends were defined as increasing (positive CI values), decreasing (negative CI values), or stationary (when the CI contained the value zero and remained constant during the study period).

To compare the risk between sexes, relative risk (RR) was calculated using the ratio between two incidences. Cases involving men were used as the numerator and those including women, as the denominator. ACL and VL RR values were calculated for 2007–2020. A 95% confidence interval was used to determine whether the RR was statistically significant. The difference between proportions was assessed using the Newcombe-Wilson method without continuity corrections.

**Figure 1:**
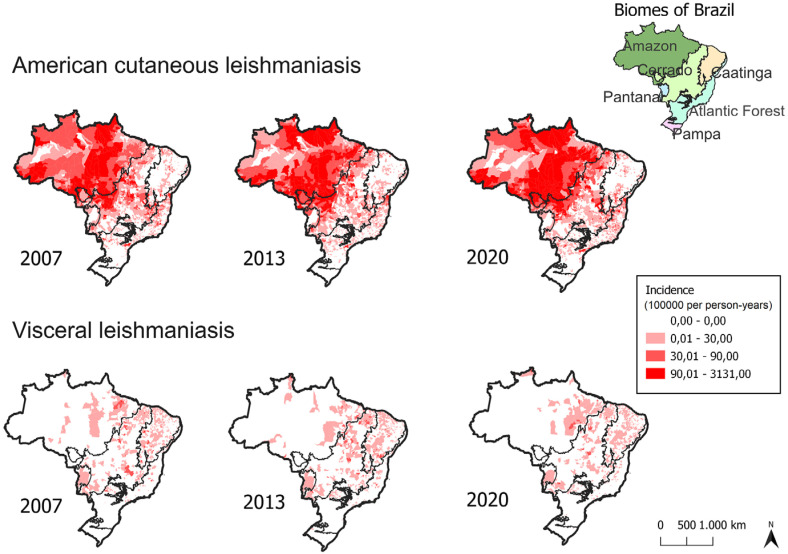
Spatial distribution of the incidence of American cutaneous leishmaniasis and visceral leishmaniasis by municipalities in Brazil in 2007, 2013, and 2020.

Incidences per municipality were calculated as the ratio of confirmed and autochthonous cases to the resident population. The data are shown as a map using the Brazilian Institute of Geography and Statistics "municipios 2022" cartographic base and the Quantum Geographic Information System 3.22 (Figure 1).

## RESULTS

During the study period, nearly all ACL (99.9%) and VL (99.2%) cases occurred in the Amazon, Atlantic Forest, Cerrado, and Caatinga. These biomes showed a decreasing temporal trend for ACL cases (Table 1). The Cerrado and Atlantic Forest biomes showed decreasing temporal trends for VL incidences, whereas the others showed stationary temporal trends for both diseases. Notably, no biomes showed an increasing temporal trend for either ACL or VL (Table 1). The annual percentage change (APC) for the decreasing temporal trend of ACL and VL varied from −0.02 to −0.06 per year (Table 1). Therefore, the study period showed subtle temporal variations in the municipal incidence of the evaluated diseases (Figure 1).

**Table 1:** Temporal trends and distribution of the incidences of American cutaneous leishmaniasis and visceral leishmaniasis by biomes in Brazil from 2007 to 2020

ACL	IR	N	RC(%)	APC	95% CI	Tendency
Amazon	51.39	138.073	55.85	−0.02	−0.02; −0.01	*Decreasing*
Atlantic Forest	4.11	59.611	24.11	−0.06	−0.09; −0.04	*Decreasing*
Cerrado	12.27	34.615	14.00	−0.03	−0.05; −0.01	*Decreasing*
Caatinga	4.91	14.727	5.96	−0.05	−0.07; −0.02	*Decreasing*
Pantanal	4.57	139	0.06	−0.15	−0.56; 0.23	Stationary
Pampa	0.07	48	0.02	−0.05	−0.10; −0.01	*Decreasing*
VL	IR	N	RC(%)	APC	95% CI	Tendency
Caatinga	4.25	12.756	34.04	−0.01	−0.02; 0.00	Stationary
Cerrado	4.29	12.084	32.25	−0.02	−0.03; −0.01	*Decreasing*
Amazon	2.55	6.844	18.26	−0.01	−0.03; 0.02	Stationary
Atlantic Forest	0.38	5.511	14.71	−0.02	−0.04; −0.01	*Decreasing*
Pantanal	7.60	231	0.62	−0.01	−0.03; 0.01	Stationary
Pampa	0.07	49	0.13	−0.01	−0.09; 0.06	Stationary

ACL = American cutaneous leishmaniasis; VL = visceral leishmaniasis; IR = incidence rate (cases/100,000 inhabitants per year); n = absolute cases in the period; RC (%) = relative cases by biome; APC = annual percentage change; 95% CI = 95% confidence interval; Tendency = trend interpretation.

Considering incidence, the Amazon (51.39 cases/100,000 inhabitants) and Cerrado (12.27 cases/100,000 inhabitants) showed the highest ACL incidence rates. The Pantanal (7.60 cases/100,000 inhabitants), Cerrado (4.29 cases/100,000 inhabitants), and Caatinga (4.25 cases/100,000 inhabitants) showed the highest incidence rates for VL (Table 1).

**Figure 2:**
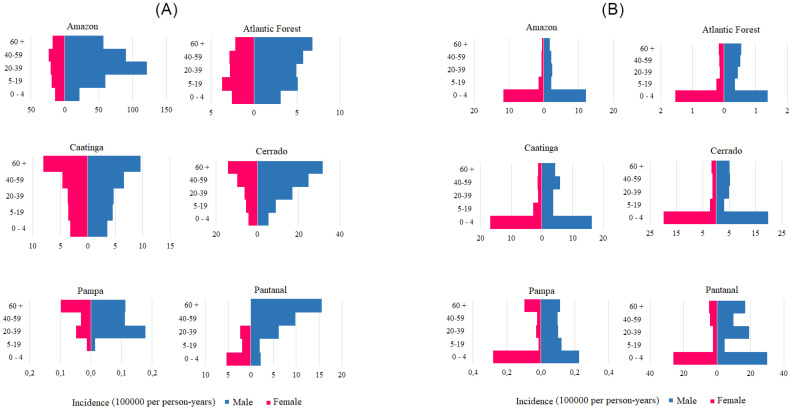
Distribution of the incidences of (A) American cutaneous leishmaniasis and (B) visceral leishmaniasis by sex, age group, and biomes in Brazil during 2007–2020.

ACL and VL incidence by sex and age group showed very different transmission profiles. ACL mostly affected older men, except in the Amazon and Pampa, in which it mainly affected men in the working age range of 20 to 39 years (Figure 2). ACL incidence RR values in men prevailed over that of women in all biomes. However, the Caatinga showed a smaller such difference (Table 2). VL mainly affected children aged from zero to four years, with a more equal ratio between boys and girls than ACL (Figure 2). However, RR prevailed in men, except for those aged from zero to four years (Table 2).

**Table 2:** Distribution of the relative risk (RR) of ACL and VL calculated by sex (male/female ratio) for age group and biome, Brazil, 2007 to 2020

ACL	Biome											
Age Group (years)	Amazon		Caatinga		Cerrado		Atlantic Forest		Pampa		Pantanal	
	RR	CI	RR	CI	RR	CI	RR	CI	RR	CI	RR	CI
≥60	3.12	2.96–3.28	1.18	1.10–1.27	2.22	2.10–2.33	3.13	2.98–3.28	11.54	3.52–37.80	2.93	1.37–6.27
40|–|59	3.80	3.69–3.91	1.43	1.34–1.53	2.57	2.46–2.67	2.01	1.95–2.08	3.36	0.91–12.42	2.31	1.23–4.31
20|–|39	5.74	5.62–5.87	1.29	1.21–1.37	2.73	2.62–2.85	1.79	1.74–1.84	3.70	1.37–9.71	2.42	1.24–4.71
5|–|19	3.04	2.97–3.12	1.26	1.18–1.35	1.63	1.54–1.73	1.38	1.34–1.43	0.96	0.06–15.43	2.25	0.60–8.48
0|–|4	1.53	1.44–1.62	1.11	0.96–1.27	1.29	1.14–1.45	1.21	1.12–1.30	-	-	1.43	0.24–8.57
**VL**	Biome											
**Age group (years)**	Amazon		Caatinga		Cerrado		Atlantic Forest		Pampa		Pantanal	
	RR	CI	RR	CI	RR	CI	RR	CI	RR	CI	RR	CI
≥60	3.08	2.30–4.12	3.41	2.95–3.94	2.89	2.52–3.32	3.30	2.77–3.93	1.15	0.35–3.77	3.56	1.62–7.83
40|–|59	3.56	2.98–4.26	4.15	3.75–4.59	3.76	3.39–4.17	3.52	3.08–4.03	4.48	0.95–21.11	2.31	1.23–4.31
20|–|39	4.05	3.51–4.61	3.00	2.74–3.28	3.86	3.53–4.23	3.32	2.92–3.76	3.48	0.78–15.56	8.33	4.22–16.43
5|–|19	1.41	1.27–1.56	1.34	1.24–1.44	1.40	1.28–1.53	1.45	1.27–1.65	8.68	1.10–68.5	1.79	0.83–3.86
0|–|4	1.04	0.97–1.12	0.96	0.90–1.02	1.00	0.94–1.06	0.90	0.81–0.99	0.79	0.24–2.61	1.15	0.73–1.81

RR = relative risk (male/female ratio); CI = Confidence interval 95%.

Regarding ACL, the Amazon had the lowest lethality rate (0.02%), with the highest percentage of cured patients (74.33%) and treatment abandonment (2.71%). In contrast, the Caatinga had the highest lethality rate (0.18%) and the lowest percentage of cured patients (65.56%) and treatment abandonment (1.03%). ACL-induced mortality showed no major differences between biomes (Table 3).

**Table 3:** Mortality, lethality, cure, and treatment abandonment rates of American cutaneous leishmaniasis and visceral leishmaniasis by biome and relative risk by sex (male/female ratio) in Brazil from 2007 to 2020

Biomes	Amazon		Caatinga		Cerrado		Atlantic Forest		Pampa		Pantanal		Total
ACL	% (n)		% (n)		% (n)		% (n)		% (n)		% (n)		n
Mortality	0.01 (31)		0.01 (26)		0.02 (60)		0.00 (69)		0.00 (0)		0.10 (3)		189
Lethality	0.02 (31)		0.18 (26)		0.17 (60)		0.12 (69)		0.00 (0)		2.16 (3)		189
Cure	74.33 (102.613)		65.56 (9.654)		70.12 (24.273)		66.44 (39.605)		66.67 (32)		72.66 (101)		176.278
Abandon	2.71 (4.887)		1.03 (169)		1.06 (494)		1.35 (1.058)		0.00 (0)		0.71 (1)		6.609
Biomes	Amazon		Caatinga		Cerrado		Atlantic Forest		Pampa		Pantanal		Total
VL	% (n)		% (n)		% (n)		% (n)		% (n)		% (n)		n
Mortality	0.13 (349)		0.28 (836)		0.30 (849)		0.03 (499)		0.01 (5)		1.02 (31)		2569
Lethality	5.10 (349)		6.55 (836)		7.03 (849)		9.05 (499)		10.20 (5)		13.42 (31)		2569
Cure	62.26 (4.401)		72.67 (8.855)		70.94 (8.194)		60.19 (4.055)		81.25 (39)		73.48 (169)		25713
Abandon	1.02 (70)		0.59 (75)		0.71 (86)		0.91 (50)		0.00 (0)		0.43 (1)		282
Biomes	Amazon		Caatinga		Cerrado		Atlantic Forest		Pampa		Pantanal		
ACL	RR	CI	RR	CI	RR	CI	RR	CI	RR	CI	RR	CI	
Mortality	1.81	0.87–3.77	2.81*	1.18–6.68	2.38*	1.37–4.14	1.98*	1.21–3.26	NC	NC	1.94	0.18–21.42	
Lethality	0.45*	0.21–0.93	2.24	0.94–5.34	1.05	0.61–1.83	1.14	0.70–1.87	NC	NC	0.80	0.07–9.12	
Cure	0.90*	0.87–0.93	0.98	0.91–1.05	1.05*	1.00–1.11	1.01	0.97–1.04	1.53	0.43–5.47	0.70	0.30–1.65	
Abandon	1.10*	1.02–1.18	1.33	0.97–1.81	1.27*	1.04–1.55	1.23*	1.08–1.40	NC	NC	0.99	0.97–1.01	
Biomes	Amazon		Caatinga		Cerrado		Atlantic Forest		Pampa		Pantanal		
VL	RR	CI	RR	CI	RR	CI	RR	CI	RR	CI	RR	CI	
Mortality	2.02*	1.62–2.53	2.12*	1.84–2.45	1.97*	1.71–2.27	2.28*	1.89–2.76	0.72	0.12–4.31	2.37*	1.09–5.16	
Lethality	1.23	0.98–1.55	1.08	0.93–1.26	1.07	0.92–1.24	1.16	0.96–1.42	0.34	0.05–2.29	1.26	0.55–2.88	
Cure	0.98	0.88–1.08	1.05	0.97–1.14	0.92*	0.85–0.99	0.86*	0.76–0.97	2.00	0.49–8.17	0.94	0.50–1.74	
Abandon	1.62	0.95–2.75	1.67	0.98–2.84	1.35	0.85–2.15	2.14*	1.07–4.29	NC	NC	1.01	0.99–1.04	

% = rate; n = number of cases; NC = no cases; ACL = American cutaneous leishmaniasis; VL = visceral leishmaniasis; RR = relative risk; CI = 95% confidence interval

* denotes significant value at the 5% level.

VL-induced mortality was 10 times higher in the Cerrado (0.3%) than in the Atlantic Forest (0.03%). Notably, the Pantanal showed high mortality (1.02%) and lethality rates (13.42%). VL cure rates between biomes varied from 60.19% to 72.67%, whereas that of treatment abandonment, from 0.59% to 1.02% (Table 3).

Except for the Amazon, all biomes showed a higher ACL-induced mortality in men. In the Amazon, being a woman constituted a protective factor against mortality and a favorable one for ACL cure. Being a man constituted a risk factor for abandoning ACL treatment in the Amazon, Cerrado, and Atlantic Forest (Table 3).

Overall, sexes showed no differences in mortality, cure, and treatment abandonment rates for VL. However, men showed a higher RR of VL-induced mortality. Being a woman constituted a protective factor in favor of VL cure in the Cerrado and Atlantic Forest (Table 3).

The study period includes 2020, a year marked by the COVID-19 pandemic. This research comparatively analyzed ACL and VL temporal trends for the pre-pandemic period (up to 2019), the results of which are presented in Supplementary Tables S1 and S2. While the trends for ACL remained unchanged, including the year 2020 showed a significant divergence for VL in the Pantanal, Cerrado, and Amazon as its historical downward trend was replaced by stability.

## DISCUSSION

Nearly all ACL and VL cases occurred in the Amazon, Atlantic Forest, Cerrado, and Caatinga. The different levels of VL incidence can be explained by an important, widely distributed vector adapted to modified environments: *Lutzomyia longipalpis*. On the other hand, a variety of *Leishmania* species, vectors, and wild reservoirs can cause ACL, resulting in a higher level of transmission, mostly in more preserved areas.

Studies in different biomes have found a dominant species of sandflies that are linked to the zoophilic and/or anthropophilic behavior of *Leishmania* vectors that can cause outbreaks in humans, leading to higher incidences of leishmaniasis^11,26,27^. Endemic areas for these diseases are associated with different specificities, such as forest cover, climatic conditions, and extensive preservation areas, favoring the preservation and diversity of sandflies^28^.

The Amazon, which is most affected by ACL, has the largest primitive vegetation cover. Thus, it shows the greatest preservation and diversity of *Leishmania* vector species that infect humans^5,8,9^.

The Atlantic Forest, Cerrado, and Caatinga are highly susceptible to environmental degradation. Therefore, changes in the environment favor the abundance of vector species and the consequent adaptation of parasites, such as VL-causing *Leishmania infantum* ^29^. Notably, VL transmission in urban areas has increased in recent decades^15,29^. Anthropization favors the expanded circulation of these parasites in metropolitan areas, such as in the state of Tocantins, which belongs to the Cerrado biome, corroborating the high incidence of VL^30^.

The Pantanal and Pampa showed much lower incidence rates than the other biomes. However, these biomes endured higher lethality rates, which may stem from a lack of access to health care services, patients discontinuing treatment, and delayed diagnosis^26^. Despite its lower incidence, the Pantanal has long been considered an area of high transmission, with a considerable number of human and canine cases, in addition to the urban adaptation of *Lu. cruzi*, an important vector, particularly in Corumba municipality^31^. Thus, although its numbers are lower when compared to other biomes, the particularities of the Pantanal make VL an extremely important disease in that area, demanding caution. The number of VL cases in the Pampa has increased in recent decades, with *Lu. longipalpis* as the main vector. However, *Pintomyia fischeri, Migonemyia migonei*, and *Lu. gaminarai* are suspected vectors in areas without *Lu. longipalpis* ^32^. Overall, the vector fauna in these regions is relatively less diverse and abundant than in other biomes.

The variation in the distribution of leishmaniasis cases between age groups and sexes indicates the possibility of extra-domestic, peridomestic, and household transmissions. Work activities in wild areas such as tourism, livestock, and agriculture result in greater exposure of working-age men to endemic areas, resulting in a greater propensity for the occupational transmission of ACL^14,33-36^. Infections in individuals aged >60 years configure a public health warning for older adults. They may be associated with occupational and leisure factors that involve activities with increased risk of leishmaniasis incidence or even indicate domestic transmission^5,14,34^. Aging leads to immunological deficiencies and comorbidities that can contribute to a lower response to the parasite^37^.

Regarding VL incidence, studies indicate that the presence of domestic and synanthropic animals and the opportunistic and eclectic eating behavior of the VL agent vector affects peri- and intra-household infection cycles; the adaptation of vectors to homes mainly affects children, women, and older adults^27,37^. With the increasing inclusion of women in the job market in rural areas, the change in the exposure profile increases their risk of infection over the years^30^.

ACL and VL incidence showed little variation throughout the study period, confirming our hypothesis regarding no increasing trend in the Brazilian biomes. The Amazon, Atlantic Forest, Cerrado, and Caatinga biomes showed a slightly decreasing temporal trend for ACL, whereas the Cerrado and Atlantic Forest showed a decrease for VL. Other biomes showed a stationary temporal trend for both diseases. The decreasing trend in the aforementioned biomes suggests the exhaustion of susceptible hosts, either because of the high incidence of infected people during the study period or changes in the territory^38^. Modified ecosystems result in less receptivity and a population decline of vectors and reservoirs of leishmaniasis. Despite a decreasing trend for ACL and VL, the occurrence of these diseases indicates a persistence of the appropriate epidemiological structure for their transmission^13,36,38^.

Notably, decreasing leishmaniasis cases requires the incorporation of preventive actions and activities by the Cutaneous and Visceral Leishmaniasis Surveillance and Control Program and the importance of maintaining active epidemiological and entomological surveillance practices in the biomes^4,5^. Associating ACL and VL cases with the spatial distribution of vectors and prioritizing areas with a higher risk of contact between vectors and humans would support more effective actions and culminate in a decreasing trend^27^.

Some studies emphasize the use by public health authorities of methods such as calculating the Global Moran Index in association with Local Spatial Association Indicators (which group areas with greater spatial similarity), highlighting trends in identifying leishmaniasis and its spatial distribution pattern^30^. Such methods can find areas with greater endemicity, direct the implementation of public policies to monitor and control diseases, and reduce the incidence of ACL and VL^27^.

The biomes with a stationary incidence trend indicated the vulnerability of prophylactic measures and control over ACL and VL cases whether due to a lack of incentives or trained professionals, difficulties in implementing prevention actions, or possible failures in surveillance actions for these diseases^13,39^. Additionally, the biota characteristics of stationary biomes may contribute to the non-reducing incidence of these diseases, along with epidemiological and entomological surveillance actions that diverge from reality^27,38^. Reportedly, despite efforts based on surveillance programs for leishmaniasis, prophylactic health actions against the transmission of ACL and VL have failed to achieve the desired effect of reducing their incidence in Brazilian territory^39^.

Cure, treatment, and abandonment coefficients showed no differences, indicating late diagnoses and the lack of prevention and control actions and conditions of access to health services^33,34^. The higher mortality in men indicates their vulnerability and predisposition to other comorbidities. The higher incidence rate in men is considered to be related to work aspects, greater resistance to healthcare services, self-care, and immediate and resolute care for the treatment of *Leishmania* infections^40^.

Notably, lethality in leishmaniasis is inherent only to early clinical and therapeutic management, and, although extremely important, may be related to patient- and biome-specific characteristics, along with the socioeconomic factors, access to health services, and health and treatment^34,36^.

Our results show a considerable decline in cases of leishmaniasis in Brazil, corroborating the technical report of the Pan American Health Organization, which found a downward trend of ACL and VL in Brazil since 2005 and 2017, respectively, and an increase of these diseases in neighboring countries^3^.

The study period included 2020, a year marked by the COVID-19 pandemic. Thus, the comparative analysis of pre- and post-pandemic data found that only VL showed a significant difference in trends. This finding contradicts the hypothesis of massive underreporting and suggests resilience in VL reporting. This phenomenon can be attributed to the increased demand for health services during the pandemic or to the rise in domestic transmission, which was favored by social isolation and the temporary suspension of vector control measures. The COVID-19 pandemic has impacted the reporting of diseases (including leishmaniasis), which showed significant differences in incidence during and after the pandemic^13^.

The limitations of this study include the use of secondary data, which may be subject to underreporting and/or incorrectly filled-out forms. Thus, they may fail to truly represent reality. Despite these limitations, the chosen data provided statistically significant information for the surveillance of temporal trends in ACL and VL in several Brazilian biomes.

Given the complexity of the assessed diseases and the environmental differences of each biome, new biome-oriented strategic approaches could successfully control leishmaniasis and formulate prevention actions since the intensity of its transmission and its risk factors may significantly vary according to local characteristics. Note that the analysis of space and time variables plays a crucial role in finding vulnerabilities, planning, and monitoring and in evaluating health actions, providing insights into where and when interventions will be most needed.

## CONCLUSION

Our results suggest that prevention and control measures cannot be based on a single strategy for all biomes since each such region has specific characteristics. The assessed diseases showed distinct transmission profiles: while ACL is mainly related to a forest and peri-urban cycle, VL is more prevalent in domestic areas. Therefore, prevention efforts against ACL should target men of productive age in the Amazon and Pampa, whereas prevention in other biomes should focus on older adults, especially male ones. For VL, all biomes should primarily direct their efforts at children under four years of age, since the vector is well adapted to the domestic environment.

We highlight that this study investigated transmission profiles according to biomes, which exceed geopolitical boundaries. Thus, the interpretation of its results must consider the ecological fallacy, which occurs when macro correlations (in this case, that regarding biomes) fail to necessarily reproduce the smaller spatial units that compose it in a homogeneous manner. Thus, we suggest the development of studies with finer meshes for more assertive prevention measures given the heterogeneity of transmission profiles.

## Data Availability

The complete anonymized dataset supporting the findings of this study is available from https://doi.org/10.48331/SCIELODATA.PFKUAQ
