# Supplementary material for: Temporal trends and spatial distribution of leishmaniasis based on biomes in Brazil, 2007 to 2020
Source: Rev Inst Med Trop Sao Paulo. 2026 Jul 20;68:e44. doi: 10.1590/S1678-9946202668044 (PMC13384505; doi:10.1590/S1678-9946202668044)
Supplement: Supplementary material [file 1678-9946-rimtsp-68-S1678-9946202668044-suppl01.pdf]

Supplementary Material of the Article "Temporal trends and spatial distribution of leishmaniasis based on biomes in Brazil, 2007 to 2020"

Corresponding Author: Andreia Fernandes Brilhante
Universidade Federal do Acre, Centro de Ciências da Saúde e do Desporto, Programa de Pós-Graduação em Ciências da Saúde na Amazonia Ocidental
E-mail: andreia.brilhante@ufac.br

Overview of the data:
This is an ecological time-series study, conducted using secondary data on cases of visceral leishmaniasis (VL) and American cutaneous leishmaniasis (ACL) registered in Brazil between 2007 and 2020. The data were obtained from TABNET DATASUS and considered stratification by Brazilian biomes, including Amazon, Caatinga, Cerrado, Atlantic Forest, Pampa, and Pantanal.

Supplementary Table S1
Temporal trends and distribution of the incidence of American cutaneous leishmaniasis (ACL) by biomes in Brazil from 2007 to 2019 and from 2007 to 2020
Collection date:
The data were collected between March 2023 and August 2023. The years of notification for the analyzed ACL cases were 2007 to 2019, and 2007 to 2020.
Description of data collection:
Data collection regarding the incidence of American Cutaneous Leishmaniasis (ACL) was carried out using secondary data obtained from the TABNET system, provided by the Department of Informatics of the Unified Health System (DATASUS).
Description of data processing methods:
Epidemiological information regarding reported cases of American Cutaneous Leishmaniasis (ACL) during the study period was selected. After data extraction from the TABNET/DATASUS system, the information was organized according to Brazilian biomes, allowing for the analysis of the spatial distribution and temporal trend of the disease in the periods 2007 to 2019 and 2007 to 2020. In the statistical analysis, trends were classified as decreasing or stationary, according to the estimated coefficients and statistical significance. The analyses were performed separately for the two investigated periods, allowing for comparison of possible changes in epidemiological trends among Brazilian biomes, including potential impacts related to the COVID-19 pandemic.

Supplementary Table S2

Temporal trends and distribution of the incidences of visceral leishmaniasis (VL) by biomes in Brazil from 2007 to 2019 and from 2007 to 2020

Collection date:

The data were collected between March 2023 and August 2023. The years of notification for the analyzed VL cases were 2007 to 2019, and 2007 to 2020.

Description of data collection:

Data collection regarding the incidence of Visceral Leishmaniasis (VL) was carried out using secondary data obtained from the TABNET system, provided by the Department of Informatics of the Unified Health System (DATASUS).

Description of data processing methods:

Epidemiological information regarding reported cases of Visceral Leishmaniasis (VL) during the study period was selected. After data extraction from the TABNET/DATASUS system, the information was organized according to Brazilian biomes, allowing for the analysis of the spatial distribution and temporal trend of the disease in the periods 2007 to 2019 and 2007 to 2020. In the statistical analysis, trends were classified as decreasing or stationary, according to the estimated coefficients and statistical significance. The analyses were performed separately for the two investigated periods, allowing for comparison of possible changes in epidemiological trends among Brazilian biomes, including potential impacts related to the COVID-19 pandemic.
