# Supplementary material for: Temporal trends and spatial distribution of leishmaniasis based on biomes in Brazil, 2007 to 2020
Source: Rev Inst Med Trop Sao Paulo. 2026 Jul 20;68:e44. doi: 10.1590/S1678-9946202668044 (PMC13384505; doi:10.1590/S1678-9946202668044)
Supplement: Supplementary material [file 1678-9946-rimtsp-68-S1678-9946202668044-suppl02.pdf]

REVISTA DO INSTITUTO DE MEDICINA TROPICAL DE SÃO PAULO

JOURNAL OF THE SÃO PAULO INSTITUTE OF TROPICAL MEDICINE

SUPPLEMENTARY MATERIAL

https://doi.org/10.48331/SCIELODATA.PFKUAQ

Temporal trends and spatial distribution of leishmaniasis based on biomes in Brazil, 2007 to 2020

Geisa Bezerra Ferreira[1], **Leonardo Augusto Kohara Melchior**[1], **Andreia Fernandes Brilhante**[1]

Supplementary Table S1 - Temporal trends and distribution of the incidences of American cutaneous leishmaniasis (ACL) by biomes in Brazil from 2007 to 2019 and from 2007 to 2020

Until 2019	β	SE	P-value	95% CI	APC	Tendency
Amazon	–0.0345	0.0089	0.003	–0.05;–0.01	–0.03	Decreasing
Caatinga	–0.0691	0.0132	0.001	–0.10;–0.04	–0.07	Decreasing
Cerrado	–0.0316	0.0118	0.021	–0.06;–0.01	–0.03	Decreasing
Atlantic Forest	–0.0496	0.0114	0.002	–0.07;–0.03	–0.05	Decreasing
Pampa	–0.1808	0.1821	0.34	–0.58; 0.22	–0.17	Stationary
Pantanal	–0.0575	0.0232	0.029	–0.11;–0.01	–0.06	Decreasing
**Until 2020**	**β**	**SE**	**P–value**	**95% CI**	**APC**	**Tendency**
Amazon	–0.0152	0.004	0.003	–0.02;–0.01	–0.02	Decreasing
Caatinga	–0.0654	0.0116	0	–0.09;–0.04	–0.06	Decreasing
Cerrado	–0.0289	0.0105	0.018	–0.05;–0.01	–0.03	Decreasing
Atlantic Forest	–0.0465	0.0104	0.001	–0.07;–0.02	–0.05	Decreasing
Pampa	–0.165	0.1834	0.386	–0.56; 0.23	–0.15	Stationary
Pantanal	–0.052	0.0216	0.033	–0.10;–0.01	–0.05	Decreasing

β = Coefficient; SE = Standard error; 95% CI = 95% confidence interval; APC = Annual percentage change; Tendency = trend interpretation.

Supplementary Table S2 - Temporal trends and distribution of the incidences of visceral leishmaniasis (VL) by biomes in Brazil from 2007 to 2019 and from 2007 to 2020

Until 2019	β	SE	P-value	95% CI	APC	Tendency
Amazon	–0.0312	0.0076	0.002	–0.05;–0.01	–0.03	Decreasing
Caatinga	–0.0654	0.0116	0	–0.10;–0.04	–0.06	Decreasing
Cerrado	–0.0289	0.0105	0.018	–0.05–0.01	–0.03	Decreasing
Atlantic Forest	–0.0465	0.0104	0.001	–0.07;–0.02	–0.05	Decreasing
Pampa	–0.165	0.1834	0.385	–0.56; 0.23	–0.15	Stationary
Pantanal	–0.052	0.0216	0.035	–0.10;–0.05	–0.05	Decreasing
**Until 2020**	**β**	**SE**	**P–value**	**95% CI**	**APC**	**Tendency**
Amazon	–0.0052	0.0119	0.669	–0.03; 0.02	–0.01	Stationary
Caatinga	–0.0148	0.007	0.055	–0.03; 0.00	–0.01	Stationary
Cerrado	–0.0226	0.0078	0.013	–0.04;–0.01	–0.02	Decreasing
Atlantic Forest	–0.0236	0.0108	0.048	–0.05;–0.00	–0.02	Decreasing
Pampa	–0.0132	0.0363	0.722	–0.10; 0.07	–0.01	Stationary
Pantanal	–0.0112	0.0121	0.373	–0.04; 0.02	–0.01	Stationary

β = Coefficient; SE = Standard error; 95% CI = 95% confidence interval; APC = Annual percentage change; Tendency = trend interpretation.

[1]Universidade Federal do Acre, Centro de Ciências da Saúde e do Desporto, Programa de Pós-Graduação em Ciências da Saúde na Amazonia Ocidental, Rio Branco, Acre, Brazil

Correspondence to: Andreia Fernandes Brilhante
Universidade Federal do Acre, Centro de Ciências da Saúde e do Desporto, Programa de Pós-Graduação em Ciências da Saúde na Amazonia Ocidental, BR 364, KM 04, Distrito Industrial, Rio Branco, AC, Brazil
Tel: +55 68 3901-2585
E-mail: andreia.brilhante@ufac.br

Received: 15 December 2025
Accepted: 13 May 2026

Editor: Maria Carmen Arroyo Sanchez

This is an open-access article distributed under the terms of the Creative Commons Attribution License.
